# Nicotinic Acid Adenine Dinucleotide Phosphate (NAADP)-mediated Calcium Signaling and Arrhythmias in the Heart Evoked by β-Adrenergic Stimulation*^♦^

**DOI:** 10.1074/jbc.M112.441246

**Published:** 2013-04-05

**Authors:** Merle Nebel, Alexander P. Schwoerer, Dominik Warszta, Cornelia C. Siebrands, Ann-Christin Limbrock, Joanna M. Swarbrick, Ralf Fliegert, Karin Weber, Sören Bruhn, Martin Hohenegger, Anne Geisler, Lena Herich, Susan Schlegel, Lucie Carrier, Thomas Eschenhagen, Barry V. L. Potter, Heimo Ehmke, Andreas H. Guse

**Affiliations:** From the Calcium Signalling Group, Departments of a Biochemistry and Signal Transduction,; b Cellular and Integrative Physiology,; e General and Interventional Cardiology,; f Medical Biometry and Epidemiology,; g Experimental Pharmacology and Toxicology, and; j Biochemistry and Molecular Cell Biology, University Medical Centre Hamburg-Eppendorf, Martinistrasse 52, 20246 Hamburg, Germany,; the c Wolfson Laboratory of Medicinal Chemistry, Department of Pharmacy and Pharmacology, University of Bath, Claverton Down, Bath BA2 7AY, United Kingdom,; the d Institute of Pharmacology, Centre for Physiology and Pharmacology, Medical University Vienna, 1090 Vienna, Austria,; h Inserm U974, Paris F-75013, France, and; the i University Pierre et Marie Curie-Paris 6, UMR-S974, CNRS UMR7215, Institut de Myologie, IFR14, F-75013 Paris, France

**Keywords:** Calcium, Calcium Intracellular Release, NAADP, Ryanodine Receptor, Signal Transduction

## Abstract

Nicotinic acid adenine dinucleotide phosphate (NAADP) is the most potent Ca^2+^-releasing second messenger known to date. Here, we report a new role for NAADP in arrhythmogenic Ca^2+^ release in cardiac myocytes evoked by β-adrenergic stimulation. Infusion of NAADP into intact cardiac myocytes induced global Ca^2+^ signals sensitive to inhibitors of both acidic Ca^2+^ stores and ryanodine receptors and to NAADP antagonist BZ194. Furthermore, in electrically paced cardiac myocytes BZ194 blocked spontaneous diastolic Ca^2+^ transients caused by high concentrations of the β-adrenergic agonist isoproterenol. Ca^2+^ transients were recorded both as increases of the free cytosolic Ca^2+^ concentration and as decreases of the sarcoplasmic luminal Ca^2+^ concentration. Importantly, NAADP antagonist BZ194 largely ameliorated isoproterenol-induced arrhythmias in awake mice. We provide strong evidence that NAADP-mediated modulation of couplon activity plays a role for triggering spontaneous diastolic Ca^2+^ transients in isolated cardiac myocytes and arrhythmias in the intact animal. Thus, NAADP signaling appears an attractive novel target for antiarrhythmic therapy.

## Teaser

**Background:** Initial studies on cardiac NAADP signaling were published, but no role for NAADP in cardiac arrhythmias has been reported.

**Results:** NAADP affects spontaneous diastolic Ca^2+^ transients in cardiac myocytes and arrhythmias in awake mice.

**Conclusion:** Results indicate a pivotal role for NAADP in fine-tuning of cardiac excitation-contraction coupling.

**Significance:** First evidence is reported for involvement of NAADP in cardiac arrhythmias evoked by β-adrenergic stimulation.

## Introduction

Nicotinic acid adenine dinucleotide phosphate (NAADP),^6^ the most powerful endogenous Ca^2+^-releasing second messenger known to date, was discovered by Lee and co-workers (1) as an impurity of commercially available NADP preparations and was structurally identified in 1995 (2). The mechanism of action of NAADP appears to involve different intracellular Ca^2+^ stores, *e.g.* acidic stores (3), nuclear envelope (4), endoplasmic reticulum (5, 6), or secretory vesicles (5). Similarly, different candidate Ca^2+^ channels have been proposed, *e.g.* members of the two-pore family (7–9), ryanodine receptors (RyRs), or transient receptor potential channels, subtype mucolipin 1 (TRP-ML1) (6, 10–16). A unifying hypothesis to integrate these different pathways of NAADP action was recently proposed (17); the central idea is that NAADP does not directly modulate channels but requires specific binding protein(s) to modulate different Ca^2+^ channels (18, 19).

Several lines of evidence support a role for NAADP in the heart as follows: (i) NAADP evoked Ca^2+^ release from heart microsomes (15); (ii) NAADP mediated activation of RyR incorporated into lipid planar bilayers (15); (iii) endogenous cardiac NAADP was detected and quantified (20, 21), and (iv) high affinity binding sites for NAADP in cardiac microsomes were observed (22). ADP-ribosyl cyclase, discussed as an enzyme involved in NAADP metabolism (23), is present in cardiac membrane preparations, and its activity is increased by stimulation of myocytes by angiotensin II or via the β-adrenergic pathway (24, 25). Further evidence for a role of NAADP in cardiac myocytes was obtained by showing that NAADP enhanced whole-cell Ca^2+^ transients and increased the amplitude and frequency of Ca^2+^ sparks (26).

In view of the strong evidence for an involvement of NAADP in cardiac Ca^2+^ signaling, we hypothesized that it might also play a significant role in aspects of myocyte function. We therefore analyzed activation of Ca^2+^ signaling upon NAADP infusion in quiescent adult mouse cardiac myocytes, and we studied both cell-based (*in vitro*) and animal (*in vivo*) models of ventricular arrhythmic events to evaluate an involvement of NAADP signaling. Our findings indicate a hitherto unappreciated pivotal role for NAADP in fine tuning of cardiac excitation-contraction coupling and open the way for novel therapeutic treatment of cardiac arrhythmias.

## EXPERIMENTAL PROCEDURES

### 

#### 

##### Materials

BZ194 was synthesized as described previously (12), purified, and checked for homogeneity by HPLC, NMR, and high resolution mass spectrometry. The specificity of BZ194 was extensively characterized both in cell culture as well as in rat (10, 12). 8-(4-Chlorophenylthio)-2′-*O*-methyladenosine-3′,5′-cyclic monophosphate (8-pCPT) and *N*^6^-benzoyladenosine-3′,5′-cyclic monophosphate (6-Bnz-cAMP) were obtained from Biolog (Bremen, Germany). Creatine kinase and creatine phosphate were purchased from Roche Applied Science. Saponin was obtained from Fluka/Sigma. Fura-2/free acid was purchased from Calbiochem. This investigation conforms to the Guide for the Care and Use of Laboratory Animals (National Institutes of Health Publication No. 85-23, revised 1985).

##### Cardiac Myocyte Isolation and Culture

Cardiac myocyte isolation from wild-type Black Swiss, FVB, or C57BL/6J mice (6–15 weeks old) and cell culture was performed as described previously (27). Hearts were digested with the perfusion buffer containing either 0.1 mg/ml Blendzyme 3 or 0.04 to 0.075 mg/ml Liberase^TM^ research grade (Roche Applied Science) and 12.5 μm CaCl_2_ for 6–9 min. A total of 150,000–250,000 rod-shaped cells were obtained per heart. Cells were plated onto laminin 111-coated (0.01 mg of laminin 111/ml, Roche Applied Science) μ-Slide 8-well chambers (Ibidi, Germany) at a density of 20,000 rod-shaped myocytes/ml in the plating medium containing minimum Eagle's medium with Hanks' salts and l-glutamine (Invitrogen), 5% (v/v) NCS or FCS, 10 mm 2,3-butanedione monoxime and incubated 30 min at 37 °C in an incubator at 2% (v/v) CO_2_ in air.

**FIGURE 1.**
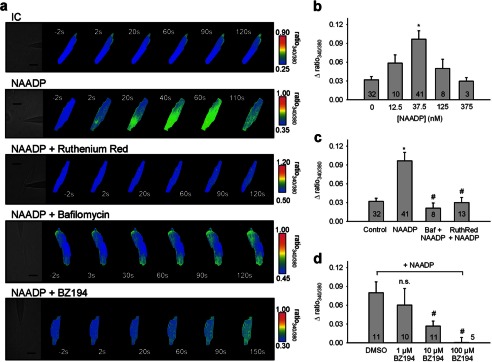
**Effects of NAADP and BZ194 on intracellular Ca^2+^ release.** *a–d,* cardiac myocytes were loaded with Fura-2/AM and subjected to combined Ca^2+^ imaging and intracellular infusion via a patch clamp pipette. *a,* change in [Ca^2+^]*_i_* is shown in pseudo-color images at different time points before and after establishing the whole-cell configuration. The position of the patch pipette can be seen in the bright field image (*scale bar,* 30 μm). Infusion of nominal Ca^2+^-free intracellular buffer did not change [Ca^2+^]*_i_*. In contrast, infusion of 37.5 nm NAADP resulted in release of Ca^2+^. Incubation with 0.5 μm bafilomycin A1 or co-infusion with 10 μm ruthenium red or 10 μm BZ194 blocked the NAADP-induced Ca^2+^ release. *IC*, nominal Ca^2+^-free intracellular buffer. *b–d,* change in [Ca^2+^]*_i_* after establishing the whole-cell configuration is summarized for the different conditions as mean ratio 340:380 ± S.E., *n* = 3–41 as indicated in the *bars. Asterisks* indicate statistical significance for NAADP compared with buffer control (*p* < 0.05). *Number signs* indicate statistical significance for NAADP plus bafilomycin A1, plus ruthenium red, or plus BZ194 compared with NAADP alone (*p* < 0.05). *n.s.* means not significant. Statistical significance *versus* control or NAADP alone was calculated by Mann-Whitney rank sum test. *d,* 0.1% (v/v) DMSO was used as control.

**FIGURE 2.**
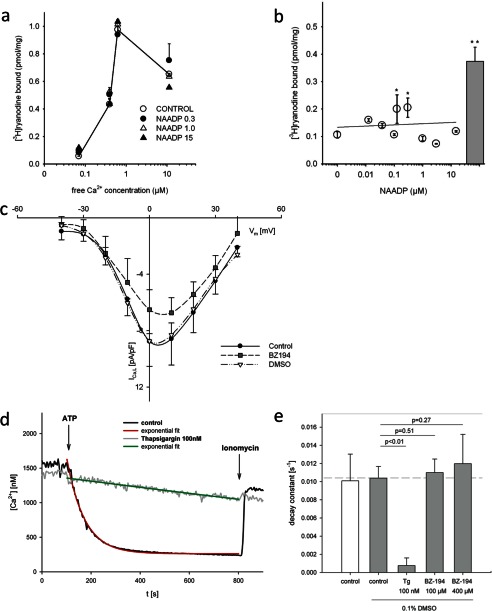
**Lack of off-target effects of BZ194 in murine ventricular cardiac myocytes.** *a,* effects of BZ194 of [^3^H]ryanodine binding to RyR2 was analyzed. Specific high affinity [^3^H]ryanodine binding to cardiac sarcoplasmic reticulum was carried out at various free Ca^2+^ concentrations in the absence and presence of NAADP (0.3, 1, and 15 μm). *Symbols* indicate the mean ± S.D. of a typical experiment, which was repeated two times. *b,* [^3^H]ryanodine binding analyzed at increasing concentrations of NAADP at 70 nm free [Ca^2+^]. *Bar* shows Ca^2+^ release by 20 mm caffeine as a positive control. Data are presented as mean ± S.E. (*n* = 2–13). *Asterisks* indicate statistical significance (*p* < 0.05). Statistical significance *versus* control was calculated for multiple comparison by analysis of variance (ANOVA) and post hoc Dunnett test. *c,* effect of BZ194 on cardiac L-type Ca^2+^ channels was analyzed in whole-cell patch clamp experiments. The current density-voltage relationship showed a statistically not significant reduction in current density, when cardiac myocytes were preincubated with BZ194 (2 mm) as compared with incubation with vehicle DMSO (0.5%, v/v). Data are presented as mean ± S.E. (*n* = 3–5). A two-factor ANOVA model with backwards selection followed by least significant difference post hoc tests was applied to investigate the effect of control, DMSO, BZ194, and different membrane potentials and their interactions on current density. *d,* effect of BZ194 on Ca^2+^ uptake in permeabilized cells was analyzed. The effect of BZ194 on Ca^2+^ uptake was analyzed in permeabilized HEK293 cells. Characteristic curves for control (*black*) and 100 nm thapsigargin (*gray*) as control for SERCA inhibition are shown. Ca^2+^ uptake was activated by addition of ATP. Curves were fitted by three parameter exponential decay (tracings in *red* and *green*, respectively). Addition of ionomycin after 800 s released Ca^2+^ from luminal space. *e,* reciprocal time constant showed no statistically significant effect, when BZ194 (100 or 400 μm) was added to HEK293 cells as compared with vehicle DMSO (0.1%, v/v). *Tg*, thapsigargin. Data are presented as mean ± S.E. (*n* = 4–10). Significant differences are indicated by *p* < 0.01, Student's *t* test.

**FIGURE 3.**
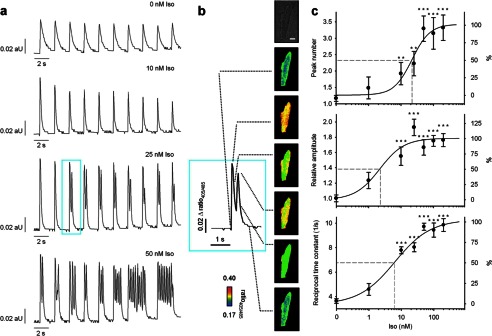
**Spontaneous diastolic Ca^2+^ transients induced by Iso.** Cardiac myocytes were loaded with indo-1/AM, and Ca^2+^ imaging was carried out as described under “Experimental Procedures.” Iso was added 5 min before recording of transients. *a,* characteristic trains of transients obtained at 0.5 s^−1^ electrical pacing are shown. *aU*, arbitrary units. *b,* enlargement of the Ca^2+^ transient from *a* is shown. The respective cell is shown in bright field. Pseudo-color images represent different time points as indicated (*dotted lines*). The number of Ca^2+^ transients per electrical stimulation, Ca^2+^ transient amplitude, and the reciprocal time constants were analyzed as a function of Iso concentration (*c*); data are presented as mean ± S.E. (*n* = 12–31). Significant differences to controls are indicated by **, *p* < 0.01, or ***, *p* < 0.001. An ANOVA model followed by LSD post hoc tests was applied to investigate the effect of different concentrations of Iso on Ca^2+^ transient amplitude and reciprocal time constant. A Mann-Whitney rank sum test was applied to investigate the effect of different concentrations of Iso on peak number.

**FIGURE 4.**
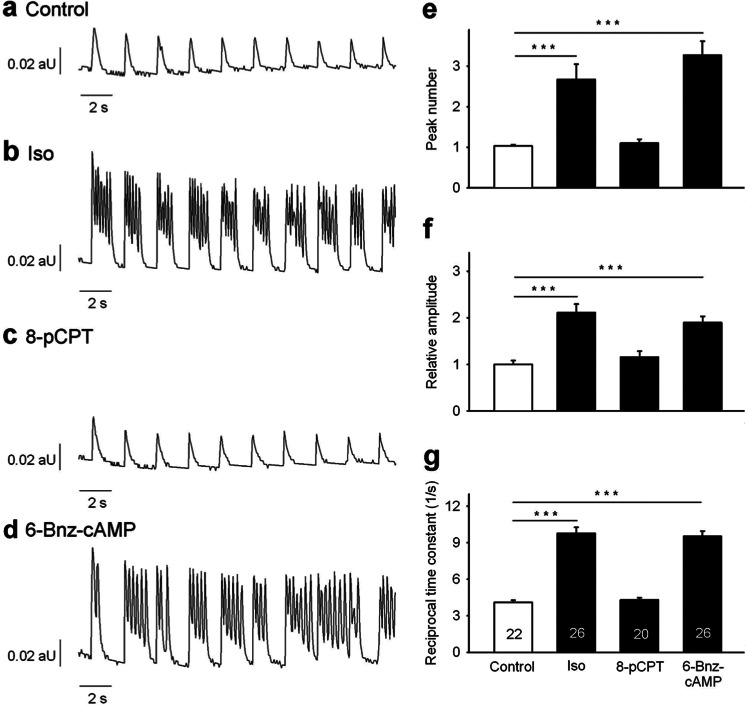
**Effect of cAMP analogs on spontaneous diastolic Ca^2+^ transients.** Cardiac myocytes were loaded with indo-1/AM, and Ca^2+^ imaging was carried out as described under “Experimental Procedures.” For activation of Epac, cells were incubated 5 min with 10 μm 8-pCPT before recording of transients. For activation of PKA, cells were incubated for 20 min with 300 μm 6-Bnz-cAMP. Because of its higher membrane permeability, 8-pCPT was used at a lower concentration and shorter incubation periods according to recent publications (49, 50). Iso (200 nm) was added 5 min before recording of transients as positive control. Untreated cells were used as negative control. Cells were electrically stimulated at 0.5 s^−1^. *a–d,* characteristic trains of transients obtained at 0.5 s^−1^ electrical pacing are shown. *aU*, arbitrary units. Peak number (*e*), amplitude (*f*), and reciprocal time constant (*g*) are presented as mean ± S.E. Significant differences are indicated by ***, *p* < 0.001, *n* = 20–26 as indicated in the *bars*. An ANOVA model followed by LSD post hoc tests was applied to investigate different effects of Iso compared with 8-pCPT and 6-Bnz-cAMP on Ca^2+^ amplitude and reciprocal time constant. A Mann-Whitney rank sum test was applied to investigate different effects of Iso compared with 8-pCPT and 6-Bnz-cAMP on peak number.

**FIGURE 5.**
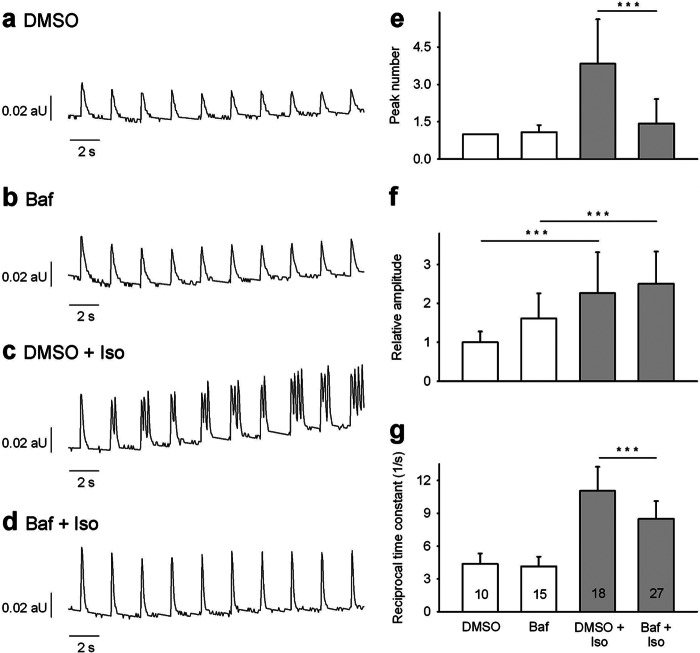
**Effect of bafilomycin A1 on spontaneous diastolic Ca^2+^ transients induced by Iso.** Cardiac myocytes were loaded with indo-1/AM, and Ca^2+^ imaging was carried out as described under “Experimental Procedures.” Cells were incubated for 20 min with 1 μm bafilomycin A1 (*Baf*) at room temperature. DMSO (0.4%, v/v) was used as control. Iso (200 nm) was added 5 min before recording of transients. *a–d,* characteristic trains of transients obtained at 0.5 s^−1^ electrical pacing are shown. *aU*, arbitrary units. Peak number (*e*), amplitude (*f*), and reciprocal time constant (*g*) are presented as mean ± S.E. Significant differences are indicated by ***, *p* < 0.001, *n* = 10–27 as indicated in the *bars*. A two-factor ANOVA model with backwards selection followed by LSD post hoc tests was applied to investigate the effect of Iso, bafilomycin A1, and their interactions on Ca^2+^ amplitude and reciprocal time constant. A Mann-Whitney rank sum test was applied to investigate the effect of Iso and bafilomycin A1 on peak number.

**FIGURE 6.**
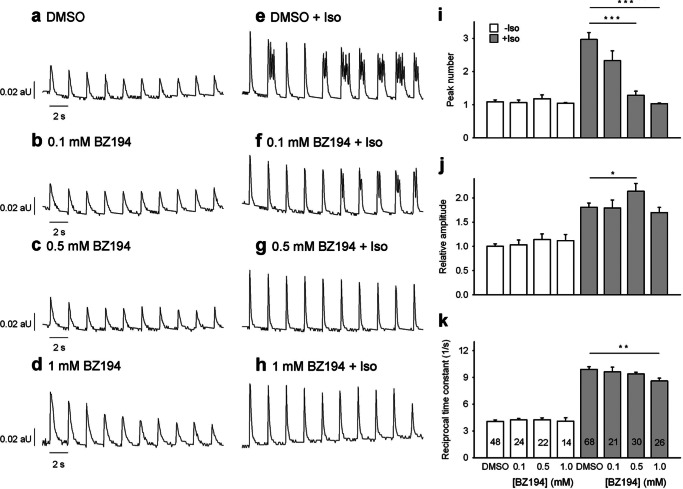
**Effect of BZ194 on spontaneous diastolic Ca^2+^ transients induced by Iso.** Cardiac myocytes were loaded with indo-1/AM, and Ca^2+^ imaging was carried out as described under “Experimental Procedures.” Cells were incubated with BZ194 for 1 h at the concentrations indicated. DMSO (0.25%, v/v) was used as control. Iso (200 nm) was added 5 min before recording of transients. *aU*, arbitrary units. *a–h,* characteristic trains of transients obtained at 0.5 s^−1^ electrical pacing are shown. Peak number (*i*), amplitude (*j*), and reciprocal time constant (*k*) are presented as mean ± S.E. Significant differences are indicated by *, *p* < 0.05; **, *p* < 0.01, or ***, *p* < 0.001, *n* = 14–68 as indicated in the *bars*. A two-factor ANOVA model with backwards selection followed by LSD post hoc tests was applied to investigate the effect of Iso, different concentrations of BZ194, and their interaction on Ca^2+^ amplitude and reciprocal time constant. A Mann-Whitney rank sum test was applied to investigate the effect of Iso and different concentrations of BZ194 on peak number.

Cardiac myocytes from Black Swiss mice were used for almost all experiments (experiments displayed in Figs. 1–4 and 6). The effect of bafilomycin A1 on spontaneous diastolic Ca^2+^ transients (SCT) was studied in cardiac myocytes from FVB mice (experiments displayed in Fig. 5). The effects of PKA- and Epac-activating cAMP analogs were studied in myocytes from Black Swiss and FVB mice (experiments displayed in Fig. 4). The effect of BZ194 was analyzed in all three mouse strains (experiments displayed in Figs. 5 and 6).

##### Whole-cell Infusion Experiments and Ca^2+^ Imaging of Cardiac Myocytes Using Fura-2

For the whole-cell infusion experiments, an EPC10 patch clamp amplifier was used in conjunction with the PATCHMASTER software (HEKA Elektronik, Lamprecht Germany). Cardiac myocytes were loaded with Fura-2/AM (4 μm final concentration) in plating medium in a reaction tube for 30 min at room temperature. Myocytes were washed twice with plating medium. For some experiments, cells were incubated with 0.5 μm bafilomycin A1 or 0.4% (v/v) DMSO for 20 min after loading with Fura-2/AM. All experiments were performed with myocytes in buffer A (containing in mm: 135 NaCl, 4.7 KCl, 1.2 MgSO_4_, 1.25 CaCl_2_, 0.6 KH_2_PO_4_, 0.6 NaH_2_PO_4_, 20 glucose, 10 HEPES, pH 7.4) attached to 35-mm glass bottom culture dishes (P35G-0-10-C MatTek, Ashland, MA) at room temperature. The patch electrodes were made from 1.5-mm diameter borosilicate glass capillaries and filled with intracellular solution. The pipette solution contained (in mm) the following: 140 KCl, 2 MgCl_2_, 10 HEPES adjusted to pH 7.4 with KOH. Fresh solution containing Fura-2 free acid (final concentration 20 μm) and NAADP (final concentrations 12.5–375 nm) were prepared each day. For co-infusion experiments, pipette solution with Fura-2, 37.5 nm NAADP, and BZ194 (final concentrations 1–100 μm), 0.1% (v/v) DMSO, ruthenium red (final concentration 10 μm), or ryanodine (1 mm) were prepared.

Ratiometric Ca^2+^ imaging was performed as described before using an imaging system (PerkinElmer Life Sciences) built around a Leica microscope (type DM IRBE) (11, 14). To reduce noise, ratio images were subjected to median filter (3 × 3) as described (28). Data processing was performed using Openlab software versions 1.7.8, 3.0.9, 3.5.2, or 5.5.2 (PerkinElmer Life Sciences).

##### Determination of Endogenous NAADP

Determination of NAADP was conducted as described in detail recently (29). In brief, hearts from wild-type mice were removed, rapidly frozen in liquid nitrogen, and homogenized in 2 ml of trichloroacetic acid (20%, w/v) using a mortar. The samples underwent two subsequent freeze-thaw cycles, using liquid nitrogen and thawing at 37 °C, and then were centrifuged (4400 × *g* for 10 min at 4 °C). The supernatant was divided into two identical halves (twin samples). Authentic NAADP (15 pmol) was added to one of the twin samples to calculate recovery. After extraction of trichloroacetic acid using water-saturated diethyl ether, the samples were freeze-dried overnight. The cell extracts were purified by gravity-fed anion-exchange chromatography, and NAADP content was determined using the NAADP cycling assay as described previously (29).

##### Staining of Lysosomes

Cells were incubated with bafilomycin A1 or DMSO (0.4% (v/v)) for 20 min at room temperature, and afterward lysosomes were labeled with 75 nm LysoTracker® Red for 20 min at room temperature. Lysosomes were visualized at excitation and emission wavelengths of 575 and 590 nm, respectively, using the PerkinElmer Life Sciences imaging systems as described for imaging of cardiac myocytes. By using a piezo-stepper at 100-fold magnification, 150 images with a distance of 200 nm were acquired in z-direction through the cell. Confocal images were obtained by off-line nearest neighbor deconvolution using the volume deconvolution module of the Openlab software as described recently (11, 30). The removal of stray light was set to 0.66, the gain to 2.45, and the offset to −336.

##### Ca^2+^ Imaging of Cardiac Myocytes Using Indo-1

Cardiac myocytes, attached to laminin 111-coated chamber slides, were loaded with indo-1/AM (4 μm final concentration) for 45 min at room temperature in plating medium (composition see above). Then the medium was removed by gentle aspiration and replaced by buffer A. This step was repeated twice to completely remove indo-1/AM as well as rounded or nonattached myocytes. Ratiometric Ca^2+^ imaging was performed as described previously (11, 14). In brief, we used a PerkinElmer Life Sciences imaging system (Tübingen, Germany) built around a Leica microscope (type DM-IRE2) at 40-fold magnification. Illumination at 355 nm was carried out using a monochromator system (Polychromator IV, TILL Photonics, Gräfelfing, Germany). The emission light beam was split using a DualView device (Improvision, Tübingen, Germany), and the individual beams were optically filtered at 405 and 485 nm. The two emission images were acquired synchronously using a grayscale CCD camera (type C4742-95-12ER; Hamamatsu, Enfield, UK; operated in 8-bit mode). The spatial resolution was 128 × 160 pixels at 40-fold magnification. The acquisition rate was ∼1 ratio/55 ms. Raw data images were stored on a hard disk. Confocal Ca^2+^ images were obtained by off-line no-neighbor deconvolution using the volume deconvolution module of the Openlab software as described recently (11, 30). The removal of stray light was set to 0.55 (removal range 0.000 (no removal) to 0.999 (highest removal)) and the gain to 4. The deconvolved images were used to construct ratio images (405:485). To reduce noise, ratio images were subjected to median filter (3 × 3) as described (28). Data processing was performed using Openlab software versions 4.0.2, 3.5.2, and 5.5.2 (PerkinElmer Life Sciences). Because of faster bleaching of fluorescence intensity at 485 nm as compared with 405 nm, fluorescence intensity ratio (405:485) increases over time. To correct for this bleaching effect in Ca^2+^ tracings, a double exponential curve was fitted to fluorescence intensity tracings. By using the equation obtained from curve fitting, a standardized ratio was calculated, allowing the original ratio to be corrected. For the stimulation protocol, electrical stimulation was achieved using a field stimulator (type SD9, Grass Technologies, West Warwick, RI) set to 30 V and custom-made platinum electrodes. Iso or 8-pCPT was added about 5 min and 6-Bnz-cAMP about 20 min before the start of electrical stimulation. Myocytes were incubated with BZ194 or vehicle DMSO (0.25%, v/v) for 1 h before the start of electrical stimulation. Myocytes were incubated with bafilomycin A1 or vehicle DMSO (0.4%, v/v) for 20 min before the start of electrical stimulation.

##### Electrophysiological Recordings of Cardiac L-type Ca^2+^ Channels

For whole-cell patch clamp experiments, an EPC9 patch clamp amplifier was used in conjunction with the PULSE stimulation and data acquisition software (HEKA Elektronik, Lamprecht Germany). The patch electrodes were pulled from 1.5 mm diameter borosilicate glass capillaries with horizontal puller (Sutter) and filled with intracellular solution. The pipette solution contained 156 mm CsCl, 1 mm MgCl_2_, and 10 mm HEPES adjusted to pH 7.2 with CsOH. Cardiac myocytes were placed in a 35-mm plastic culture dish and adhered loosely in extracellular buffer B (containing in mm: NaCl 140, KCl 5, MgCl_2_ 2, CaCl_2_ 2, glucose 10, HEPES 10, pH 7.3). The experiments were either performed without incubation (control), after incubation of the myocytes in 0.5% (v/v) DMSO (37 °C, 1 h), or after incubation with 2 mm BZ194 (37 °C, 1 h). The holding potential for the measurements of L-type Ca^2+^ channels was −80 mV. A prepulse to −40 mV for 100 ms was applied to inactivate Na^+^ channels. IV relationships were recorded from −40 to +40 mV. Series resistance was compensated by 70–90%. Data were low pass filtered at 1 kHz and stored on a personal computer. Analysis was performed with PulseFit software (HEKA) and Excel (Microsoft). All experiments were performed at room temperature.

##### High Affinity [^3^H]Ryanodine Binding

Cardiac heavy sarcoplasmic reticulum was prepared from rabbit hearts according to a previous protocol (31). High affinity [^3^H]ryanodine binding was carried out with 50 μg of heavy SR, which was incubated for 3 h at 30 °C in a buffer containing (in mm) 20 HEPES, pH 7.4, 140 KCl, 50 NaCl, and 20 nm [^3^H]ryanodine supplemented by protease inhibitors (1 μm leupeptide, 1 μm aprotinin, 10 μm calpain inhibitors I and II, and 100 μm 4-(2-aminoethyl)benzenesulfonyl fluoride hydrochloride, Pefabloc SC). The free [Ca^2+^] was adjusted by the ratio of CaCl_2_ and EGTA (32). The binding reaction was terminated by filtration. Specific binding was determined by subtraction of nonspecific binding in the presence of 20 μm ryanodine.

##### Determination of SERCA Activity in Permeabilized Cells

HEK293 cells (1·10^7^) were rinsed twice in intracellular buffer I (containing in mm: 10 NaCl, 120 KCl, 1.2 MgCl_2_, 0.533 CaCl_2_, 1 mm EGTA, 10 HEPES, pH 7.2, and free [Ca^2+^] of this solution was 170 nm (calculated with MaxChelator)). Afterward, cells were resuspended in permeabilization buffer (intracellular buffer I with 60–80 μg/ml saponin) and incubated for 5 min at 37 °C. After incubation, cells were rinsed with intracellular buffer II (containing in mm: 10 NaCl, 120 KCl, 1.2 MgCl_2_, 10 HEPES, pH 7.4, and free [Mg^2+^] of this solution is 300 nm after addition of 1 mm ATP (calculated with MaxChelator) and resuspended in intracellular buffer II). To measure free [Ca^2+^], 0.5 μg/ml Fura-2/free acid, 20 units/ml creatine kinase, and 20 mm creatine phosphate were added to the cells in a quartz cuvette. Fura-2 fluorescence at 495 ± 10 nm was determined in a Hitachi F-2000 fluorescence spectrophotometer (Colora Messtechnik, Lorch, Germany) with alternating excitation at 340 ± 10 and 380 ± 10 nm every 5 s. After 100 s, 1 mm ATP was added to activate Ca^2+^ uptake. After 800 s, 1 μm ionomycin was added to release Ca^2+^ from the luminal space. Each measurement was calibrated according to Ref. 33 using 2 mm CaCl_2_ for maximal ratio and 8 mm EGTA, 60 mm Tris for minimal ratio. Curves after addition of ATP were fitted to three-parameter exponential decay using SigmaPlot (10.0).

##### Action Potential Recordings

Action potentials of cardiac myocytes were recorded using the ruptured patch whole-cell configuration in combination with an EPC-10 amplifier controlled by the PATCHMASTER software. Patch pipettes were pulled from 1.5-mm diameter borosilicate glass capillaries with a horizontal puller (Sutter Instruments). The pipette solution contained (mm) 140 KCl, 2 MgCl_2_, 1 CaCl_2_, 2.5 EGTA, 10 HEPES, titrated to pH 7.40 using KOH. The extracellular solution consisted of (mm) 135 NaCl, 4.7 KCl, 0.6 KH_2_PO_4_, 1.2 MgSO_4_, 0.6 NaH_2_PO_4_, 1.25 CaCl_2_, 20 glucose, 10 HEPES, titrated to pH 7.4 with NaOH. Before beginning the experiments, myocytes were incubated for 1 h with BZ194 (2 mm, dissolved in 0.5% DMSO) or vehicle DMSO (0.5%). Action potentials were elicited at room temperature at a rate of 2 Hz by depolarizing current pulses of 5 ms duration throughout the experiment. The current injection was adapted to match the excitation threshold of each individual myocyte. Data were low pass filtered at 1 kHz and analyzed using the FitMaster software (version 2.x60, Heka Elektronik) and custom-made procedures in IgorPro (version 6.2, Wavemetrics, Lake Oswego, OR).

##### Imaging of SR-Luminal Ca^2+^ Concentration in Cardiac Myocytes Using mag-Fura-2

Cardiac myocytes were loaded with mag-Fura-2/AM (5 μm final concentration) in plating medium (composition see above) in a reaction tube and attached to laminin 111-coated chamber slides for 30 min at 37 °C in an incubator at 2% (v/v) CO_2_ in air. To remove mag-Fura-2/AM and nonattached myocytes, the chamber slides were washed with buffer A (composition see above). The myocytes were then incubated with 1 mm BZ194 or vehicle DMSO (0.25%, v/v) for 60 min at 37 °C and 2% (v/v) CO_2_ in air. Iso (200 nm, fresh solution) was added 5 min before imaging.

Ca^2+^ imaging was performed as described above using a PerkinElmer Life Sciences imaging system built around a Leica microscope (type DM IRBE). Illumination at 380 nm was carried out using a Sutter DG-4 ultra high speed wavelength device (PerkinElmer Life Sciences). Images of emission light at 510 nm were acquired using a gray scale EM-CCD camera (type C9100-02; Hamamatsu; operated in 14-bit mode). The spatial resolution was 1000 × 1000 pixels at 40-fold magnification. The acquisition rate was ∼30 frames/s. Raw data images were stored on hard disk. Data processing was performed using Openlab software version 5.5.2 (PerkinElmer Life Sciences).

##### Stimulation Protocol

Myocytes were monitored under repeated electrical stimulation (5 times, 0.5 s^−1^) and subsequent addition of caffeine (10 mm) using a field stimulator (type SD9, Grass Technologies, West Warwick, RI) set to 30 V and custom-made platinum electrodes.

##### In Vivo β-Adrenergic Provocative Testing

For *in vivo* β-adrenergic provocative testing, telemetric ECG transponders (ETA-F10, Data Sciences International, St. Paul, MN) were implanted subcutaneously in male BALB/c mice (24.7 ± 0.5 g, *n* = 6) with the leads approximating an Eindhoven II configuration (34). Postoperative care, including analgesic and antibiotic treatment, followed institutional guidelines. Two weeks after surgery, mice were subjected to β-adrenergic provocative testing. Each mouse underwent all four β-adrenergic provocative testings at a random order with an interval of 1 week between the tests. Four hours prior to β-adrenergic provocative testing, mice were intraperitoneally injected with the following: (i) NaCl (1 ml/kg, 1% serum); (ii) DMSO (1 ml/kg, 1% serum), or (iii) BZ194 (180 mg/kg, 1 ml/kg DMSO, 1% serum). The mice were then challenged by two subsequent intraperitoneal injections of either NaCl (0.9%, w/v) or Iso (2 mg/kg). The ECG was continuously recorded throughout the experiment at a sampling rate of 1 kHz using Dataquest A.R.T (version 4.0, Data Sciences International). ECGs were analyzed semi-automatically with ECG auto (version 2.5.1.18, emka Technologies, Paris, France) using animal-specific waveform libraries as well as automatic RR-interval detection. Detection gaps were manually evaluated. Arrhythmias were classified according to The Lambeth Conventions (35) by an operator blinded to the protocol.

##### Analysis and Statistics

Amplitudes obtained from Ca^2+^ imaging of cardiac myocytes were normalized to the mean amplitude of the control per day. Significance tests applied are mentioned in the figure legends. Level of significance was set to *p* < 0.05, two sided. Calculations were done using SPSS (version 18.0.0) and Prism (versions 4 and 5; GraphPad).

## RESULTS

### 

#### 

##### Ca^2+^ Mobilizing Activity of NAADP in Cardiac Myocytes

To analyze the Ca^2+^ mobilizing activity of NAADP in ventricular cardiac myocytes, cells were infused with 37.5 nm NAADP resulting in an increase in [Ca^2+^]*_i_*, whereas infusion of nominally Ca^2+^-free intracellular buffer did not (Fig. 1*a*, *2nd versus 1st row*). A slow wave of increased [Ca^2+^]*_i_* originated from the tip of the patch pipette (Fig. 1*a*, *2nd row*). Different concentrations of NAADP revealed a bell-shaped concentration-response curve with a maximum around 37.5 nm NAADP (Fig. 1*b*). Further evidence for a physiological role of NAADP in cardiac myocytes was obtained by determining endogenous NAADP concentration in extracts of whole hearts freshly prepared using an enzymatic assay with femtomole sensitivity (29), revealing a concentration of 553 ± 244 fmol/mg protein (mean ± S.E.; *n* = 3), a value well in accordance with data recently obtained in mouse heart (20).

As it has been established in different cell types that either NAADP activates RyR (12, 13) or Ca^2+^ released by NAADP amplifies Ca^2+^ release via d-myo-inositol 1,4,5-trisphosphate receptors or RyRs (7, 8, 36), we tested an involvement of RyRs. Incubation of cardiac myocytes with ruthenium red fully inhibited NAADP-mediated Ca^2+^ signaling, indicating involvement of RyRs (Fig. 1, *a, 3rd row,* and *c*). Furthermore, ryanodine also fully inhibited Ca^2+^ signals evoked by NAADP; whereas infusion of pipette solution (vehicle) resulted in a Fura-2 ratio of 0.912 (*n* = 2; mean), NAADP increased the Fura-2 ratio to 1.067 ± 0.033 (mean ± S.E., *n* = 5). Co-infusion of NAADP and ryanodine resulted in a Fura-2 ratio of 0.876 ± 0.034 (mean ± S.E., *n* = 4; *p* = 0.005 *versus* NAADP alone, *t* test). Infusion of ryanodine alone resulted in a Fura-2 ratio of 0.900 ± 0.024 (mean ± S.E., *n* = 3), respectively. Besides the endoplasmic reticulum, acidic Ca^2+^ stores have been postulated to be involved in NAADP signaling in different cell types (3, 26, 36, 37). To analyze this, cardiac myocytes were incubated with bafilomycin A1, an inhibitor of the proton pump of acidic stores. Preincubation with bafilomycin A1 prevented the characteristic punctate staining pattern obtained with Lysotracker® Red, indicating breakdown of proton and Ca^2+^ gradients across the membrane of the acidic compartment (data not shown). Incubation of cardiac myocytes with bafilomycin A1 completely inhibited NAADP-mediated Ca^2+^ signaling, indicating that acidic stores are involved in the process of NAADP signaling in cardiac myocytes (Fig. 1, *a, 4th row,* and *c*).

BZ194 (3-carboxy-1-octylcarbamoylmethylpyridinium bromide) is a specific antagonist of NAADP characterized in detail recently, and when applied directly on target, BZ194 inhibited NAADP-mediated Ca^2+^ signaling without any inhibitory effect on Ca^2+^ release induced by d-myo-inositol 1,4,5-trisphosphate or cyclic ADP-ribose (12). In cardiac myocytes, infusion of BZ194 antagonized Ca^2+^ mobilization induced by NAADP in a concentration-dependent manner reaching complete blockade at 10 μm (Fig. 1, *a, 5th row,* and *d*). These experiments in quiescent cardiac myocytes indicate a potential involvement of NAADP and acidic Ca^2+^ stores in RyR modulation during cardiac Ca^2+^ signaling.

The sensitivity of NAADP-evoked Ca^2+^ signaling to bafilomycin A1, ruthenium red, and ryanodine opens the possibility for acidic stores or RyR as direct NAADP targets. Thus, we analyzed a potential direct modulation of RyR. [^3^H]Ryanodine binding to cardiac heavy SR depended strongly on the free [Ca^2+^], although NAADP added at concentrations of >300 nm did not modulate [^3^H]ryanodine binding (Fig. 2*a*). Although a small but significant stimulatory effect of NAADP was detected at 125 and 300 nm NAADP, this was negligibly small when compared with the prototypical RyR activator caffeine (Fig. 2*b*), indicating minor activation of RyR2 by NAADP.

Potential unspecific effects of BZ194 were assessed as follows. L-type Ca^2+^ currents were almost unaffected by BZ194 even at a high concentration of 2 mm (Fig. 2*c*). SERCA activity was analyzed in permeabilized HEK293 cells upon ATP addition. The permeabilized cell preparation allowed BZ194 to directly act on any potential intracellular target (Fig. 2*d*). Addition of BZ194 (up to 400 μm) did not affect the rate of Ca^2+^ uptake, although SERCA inhibitor thapsigargin did (Fig. 2, *d* and *e*). Thus, neither L-type Ca^2+^ currents nor SERCA activity was impaired by BZ194, further supporting the specificity of the NAADP-antagonist BZ194.

##### Spontaneous Diastolic Ca^2+^ Transients upon β-Adrenergic Stimulation

To analyze Ca^2+^ signaling upon β-adrenergic stimulation, myocytes were electrically stimulated at 0.5 s^−1^. The vast majority (>80%) of control cells (addition of solvent only) responded with a single characteristic Ca^2+^ transient to electrical pacing (Fig. 3, *a* and *c, upper panel*). In the presence of 10 nm Iso, cells also showed regular single Ca^2+^ transients (Fig. 3*a*, *1st* and *2nd row*). 10 nm Iso increased peak amplitude by ∼70% and accelerated transient decay as compared with control (Fig. 3*c*, *middle* and *lower panels*). Increasing Iso to 25 nm started to induce SCT (Fig. 3, *a, 3rd* and *4th row,* and *b*) showing a clear concentration-dependent effect with an EC_50_ of about 22 nm (Fig. 3*c*, *upper panel*). Increases in amplitude and reciprocal time constant were already observed with EC_50_ of about 2 and 6 nm, respectively, suggesting the involvement of different signaling mechanisms or different signaling thresholds downstream of β-adrenoceptors.

Because SCT occurred upon strong β-adrenergic stimulation, it was likely that cAMP generation and its downstream effectors PKA or “exchange protein activated by cAMP” (Epac) might be involved. Indeed, the PKA-selective cAMP-analog 6-Bnz-cAMP (38) induced SCT (Fig. 4, *d* and *e*) in a fashion similar to high Iso concentrations (Fig. 4, *b* and *e*), whereas the Epac-selective cAMP analog 8-pCPT (39) did not (Fig. 4, *c* and *e*). Furthermore, only 6-Bnz-cAMP induced a marked increase of amplitude and reciprocal time constant, again mimicking the effect of Iso (Fig. 4, *f* and *g*).

##### Effect of Bafilomycin A1 on SCT Induced by β-Adrenergic Stimulation

To investigate involvement of acidic stores in SCT, myocytes were preincubated with bafilomycin A1. In the absence of Iso, bafilomycin A1 had no significant effect on SCT numbers, Ca^2+^ transient amplitude, or reciprocal time constant (Fig. 5, *a*, *b*, and *e–g*). In stark contrast, preincubation with bafilomycin A1 almost completely blocked all SCT induced by high Iso, with the majority of cells showing only one Ca^2+^ transient per electrical stimulation (Fig. 5, *d versus c* and *e*). However, bafilomycin A1 had no impact on Iso-stimulated Ca^2+^ amplitude (Fig. 5*f*) but induced a small, significant reduction of the reciprocal time constant from 11 to 8.6 s^−1^ (Fig. 5*g*).

##### BZ194 Blocks SCT Induced by β-Adrenergic Stimulation

Having observed the strong reduction of SCT upon bafilomycin A1 treatment, we hypothesized that NAADP might be the endogenous stimulus for SCT. Because we directly demonstrated the inhibitory effect of BZ194 in quiescent cardiac myocytes infused with NAADP (Fig. 1), cardiac myocytes were preincubated with BZ194, and [Ca^2+^]*_i_* was imaged at constant electrical stimulation in the presence and absence of Iso. A concentration-dependent decrease of Iso-induced SCT was obtained upon preincubation with BZ194, whereas Ca^2+^ transient amplitudes were almost unchanged (Fig. 6). Although BZ194 had no effect on Ca^2+^ transients in the absence of Iso (Fig. 6, *a–d* and *i–k*), BZ194 ≥0.5 mm significantly reduced the number of SCT evoked by Iso (Fig. 6, *e–k*). At 1 mm BZ194, only one transient per electrical stimulation was observed (Fig. 6, *h* and *i*). At this concentration, BZ194 also induced a small but significant decrease of the reciprocal time constant from 10 to 8.5 s^−1^ (Fig. 6*k*).

**FIGURE 7.**
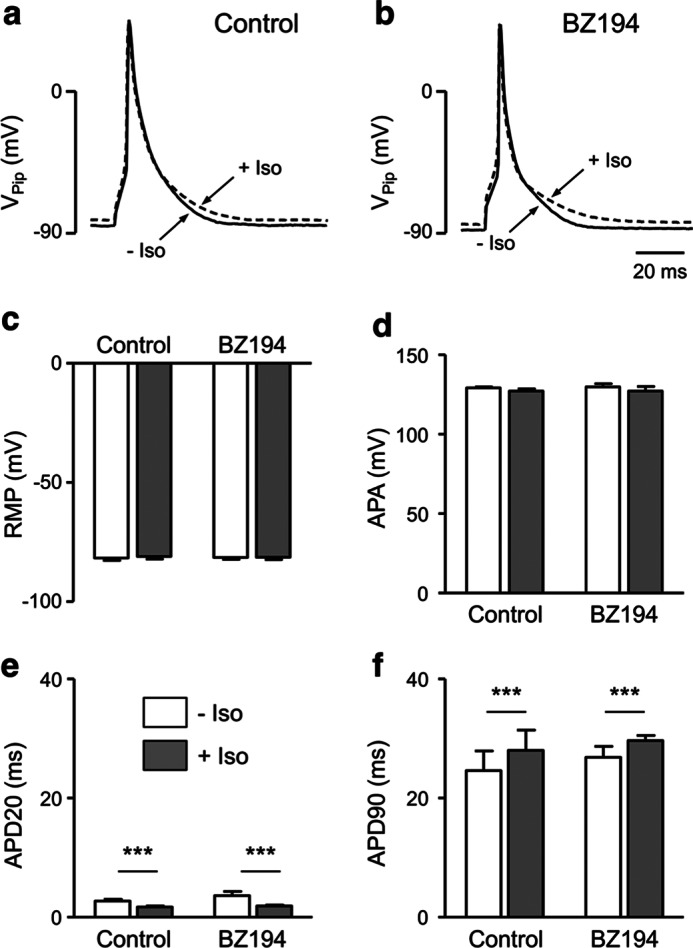
**Effects of BZ194 on action potentials of murine ventricular cardiac myocytes.** The effect of BZ194 on action potentials was analyzed in ventricular cardiac myocytes isolated from mice using whole-cell patch clamp experiments. *a* and *b,* representative action potentials recorded from an untreated cardiac myocyte (*a, control*) and from a cardiac myocyte preincubated with 2 mm BZ194 (*b*) in the absence (−*Iso*) and presence of 200 nm Isol (+*Iso*). *c–f*, mean (± S.E.) action potential characteristics calculated from action potential recordings obtained from untreated (*n* = 11, vehicle) and BZ194-treated (*n* = 6) cardiac myocytes as shown in *a* and *b. RMP*, resting membrane potential; *APA*, action potential amplitude; *APD20*, action potential duration at 20% repolarization; *APD90*, action potential duration at 90% repolarization. ***, *p* < 0.001 repeated measures ANOVA for paired experiments.

To address the possibility that BZ194 might directly affect cardiac plasma membrane currents, its effect on action potentials was assessed in isolated cardiac myocytes. Action potentials rely on a finely tuned interaction between all currents and thus represent a very sensitive readout for off-target effects. Neither in the absence (Fig. 7*a*) nor in the presence of Iso (200 nm, Fig. 7*b*) did BZ194 affect the shape or the duration of the action potentials. In particular, the resting membrane potential (Fig. 7*c*), the action potential amplitude (Fig. 7*d*), and the time course of early and late repolarization (Fig. 7, *e* and *f*) were unaffected. This makes a direct effect of BZ194 on currents highly unlikely, *e.g.* the inward rectifying K^+^ current, the fast Na^+^ current, the L-type Ca^2+^ current (see also Fig. 2*c*), or the repolarizing K^+^ currents. Taken together, the results indicate that the decrease of SCTs by BZ194 is not a consequence of direct effects on these cardiac membrane currents.

##### Effect of BZ194 on Sarcoplasmic Reticular-Luminal Ca^2+^ Concentration during Electrical and β-Adrenergic Stimulation

**FIGURE 8.**
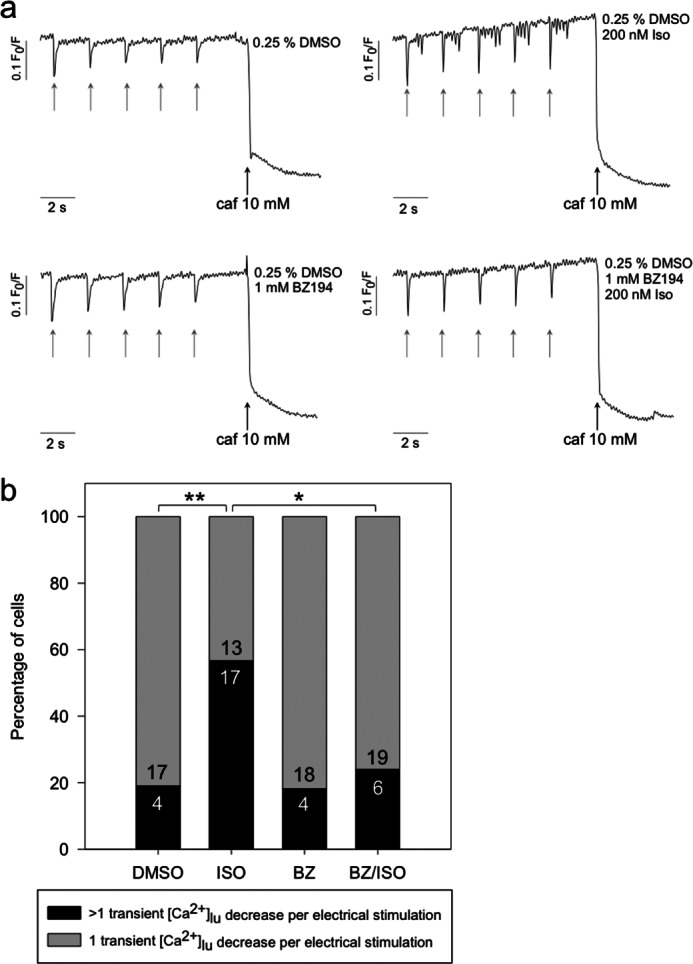
**Effect of BZ194 on sarcoplasmic reticular-luminal Ca^2+^ concentration ([Ca^2+^]_SR_) during electrical and β-adrenergic stimulation.** Murine ventricular cardiac myocytes were loaded with 5 μm mag-Fura-2/AM for 30 min at 37 °C and 2% CO_2_ (v/v). Time for uptake into the SR and hydrolysis of the ester and incubation with either 0.25% vehicle DMSO (control) or 1 mm BZ194 (*BZ*) was 60 min at 37 °C and 2% CO_2_ (v/v). Incubation time with 200 nm isoproterenol was 5 min directly before the measurement. Ca^2+^ imaging was carried out as described under “Experimental Procedures.” Fluorescence at 380 nm was measured, and the ratio of *F*_0_/*F* was calculated. *a,* typical transients obtained at 0.5 s^−1^ electrical pacing (*gray arrows*) and subsequent addition of 10 mm caffeine (*caf*) (*black arrows*) are shown. *b* shows the percentage of cells with only one transient [Ca^2+^]_lu_ decrease *versus* cells showing more than one transient [Ca^2+^]_lu_ decrease. *Numbers within the bars* represent the sample size. *Asterisks* denote statistical significant differences (**, *p* = 0.0081; *, *p* = 0.0158; Mann-Whitney rank sum test).

Ventricular myocytes loaded with mag-Fura-2 showed single transient decreases of the sarcoplasmic reticular-luminal Ca^2+^ concentration ([Ca^2+^]_SR_) upon electrical stimulation at 0.5s^−1^ (Fig. 8*a*). Under control conditions (cells preincubated with vehicle DMSO), the transient decreases of [Ca^2+^]_SR_ showed a relatively slow recovery back to base-line values, thus resembling the slow decay of cytosolic Ca^2+^ transients under control conditions (Fig. 3*a*). 81% of cardiac myocytes displayed one transient decrease of [Ca^2+^]_SR_ at each single electrical stimulation (Fig. 8*b*). Transient decreases in [Ca^2+^]_SR_ in cardiac myocytes preincubated with BZ194 were of similar shape as compared with controls (Fig. 8*a*); moreover, a similar percentage of cells displaying one transient decrease of [Ca^2+^]_SR_ at each single electrical stimulation was observed when compared with vehicle DMSO (Fig. 8*b*). Strong β-adrenergic stimulation influenced both the shape and number of transient decreases in [Ca^2+^]_SR_ (Fig. 8, *a* and *b*). The recovery back to base line of the transient decreases in [Ca^2+^]_SR_ proceeded more rapidly as compared with controls (Fig. 8*a*), again resembling the changes in shape of cytosolic Ca^2+^ transients evoked by Iso (Figs. 8*a versus* 3*a*). Importantly, spontaneous diastolic transient decreases in [Ca^2+^]_SR_ (SDC) were observed upon strong β-adrenergic stimulation (Fig. 8*a*). The percentage of myocytes displaying SDC significantly increased from 19% (DMSO) to 57% (Fig. 8*b*). Importantly, preincubation of myocytes with BZ194 and subsequent strong β-adrenergic stimulation resulted in a significant decrease in the percentage of cells displaying SDC to 24% (Fig. 8*b*). At the end of each experiment, 10 mm caffeine was added to deplete the SR (Fig. 8*a*). Taken together, the data indicate that strong β-adrenergic stimulation resulted in spontaneous diastolic transient decreases in [Ca^2+^]_SR_ (SDC), most likely due to repetitive opening of sensitized RyR2.

##### BZ194 Reduces Arrhythmic Events in Vivo

**FIGURE 9.**
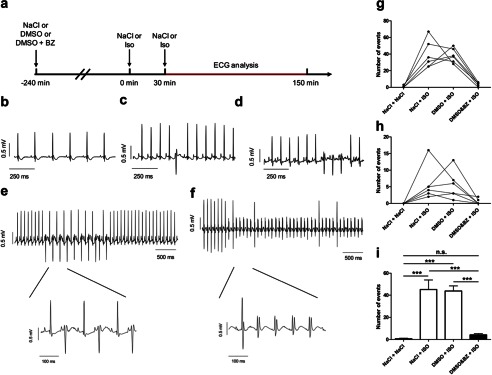
**Effect of BZ194 on arrhythmia induction *in vivo*.** *a,* mice (*n* = 6) were challenged by two consecutive injections (30 min apart) of NaCl or Iso (2 mg/kg) 4 h after injection of either NaCl, DMSO (1 mg/kg), or DMSO + BZ194 (*BZ*) (180 mg/kg). *b,* representative ECG tracing following an injection of BZ194 (180 mg/kg) alone. Representative recordings of arrhythmias were evoked by the injection of Iso including a premature ventricular beat (PVB, *c*), salve of PVBs (*d*), bigeminy (*e*), and VT (*f*). Effect of NaCl, Iso, DMSO, and BZ194 on the occurrence of (*g*) PVBs and bigeminy, and (*h*) salves of PVBs and VTs. *Lines* depict responses in individual mice. *i,* summary analysis of the total number of arrhythmic events. Significant differences are indicated by ***, *p* < 0.001, ANOVA followed by a paired Bonferroni's post hoc test; *n.s.*, not significant.

To determine whether the observed inhibitory properties of BZ194 on alterations in myocardial Ca^2+^ signaling by β-adrenergic stimulation translate into protection against arrhythmias in the intact organism, we investigated the consequences of Iso on cardiac electrophysiology with or without administration of BZ194 (180 mg/kg body weight) in awake mice (Fig. 9*a*). Administration of BZ194 induced a prominent but transient tachycardia that reached its maximum ∼60 min after injection and then slowly faded. BZ194 had no effect on the morphology of the ECG (Fig. 9*b*) and did not cause arrhythmic events in any animal. Four hours later, all animals received two intraperitoneal injections of Iso (2 mg/kg body weight) within 30 min, and ECGs were recorded for 120 min after the last injection (Fig. 9*a*). Iso rapidly increased heart rate and induced a variety of arrhythmias, including premature ventricular beats (PVB) (Fig. 9*c*), salves of PVBs (Fig. 9*d*), bigeminy (Fig. 9*e*), and ventricular tachycardia (VT, Fig. 9*f*). Ventricular fibrillation was not observed. Each animal developed arrhythmias after Iso; ∼90% of the observed events were PVBs (Fig. 9, *g* and *h*). DMSO, used as a solvent for BZ194, did not affect the occurrence of any of the arrhythmic events (Fig. 9, *g* and *h*) evoked by Iso. In contrast, when BZ194 was injected 240 min prior to the first dose of Iso, the frequency of PVBs and bigeminy was reduced by 91 ± 2% (Fig. 9*g*) and that of salves of PVBs and VTs was reduced by 88 ± 11% (Fig. 9, *h* and *i*). The number of events observed after combined Iso and BZ194 treatment was not significantly different from controls (Fig. 9*i*). After completion of the *in vivo* β-adrenergic provocative testing, all mice were monitored over the following week. All animals, except for those treated with Iso in combination with BZ194, recovered well with no signs of physical or behavioral impairment. In contrast, mice treated with Iso and BZ194 showed a reduced motor activity and an impaired general condition, and three of the six animals died 1–4 days after β-adrenergic provocative testing.

## DISCUSSION

Here, we describe a novel mechanism involved in catecholamine-induced arrhythmias in mouse heart. NAADP, the most potent Ca^2+^-releasing messenger known to date, induced Ca^2+^ signaling in quiescent cardiac myocytes, a process sensitive to antagonists of RyRs and NAADP signaling and to inhibitors of H^+^-ATPase of acidic stores. Furthermore, SCT observed in the presence of high concentrations of Iso in electrically driven myocytes were sensitive to both inhibition of Ca^2+^ loading of acidic stores and NAADP antagonism. Spontaneous Ca^2+^ release events were directly observed by live cell imaging of both [Ca^2+^]*_i_* and [Ca^2+^]_SR_. Our data suggest that NAADP-induced Ca^2+^ release from (micro)domains, *e.g.* acidic stores, close to SERCA and/or RyRs increases the sensitivity of the RyR. The sensitized Ca^2+^ release system in turn leads to SCT even in the absence of the regular trigger, Ca^2+^ entry via L-type Ca^2+^ channels. The observation that the NAADP antagonist BZ194 largely ameliorated Iso-induced arrhythmias in awake mice provides strong evidence that NAADP-mediated modulation of couplon activity plays a pivotal role for triggering arrhythmias in the intact animal. Importantly, because systolic Ca^2+^ transients were not affected regarding transient amplitude and only slightly regarding reciprocal time constant, NAADP signaling may become an attractive target for antiarrhythmic therapy.

NAADP has been proposed to act via RyRs (4, 12, 13), TRP-ML1 (16), or two-pore channels (7, 8). Evidence for a role of NAADP in heart is emerging, as detailed in the Introduction (15, 20–26); however, others reported a lack of effect of NAADP in heart (40). The latter study was conducted using SR microsomes or RyR incorporated into lipid planar bilayers (40). Possibly, the NAADP-sensitive compartment in cardiac myocytes, acidic stores, was depleted in the SR microsome fraction used and thus may explain the negative results.

Many studies found that the Ca^2+^ releasing activity of NAADP was sensitive to a functional H^+^ gradient across the membranes of acidic stores. Maneuvers resulting in a breakdown of the H^+^ gradient also destroyed the secondary gradient of Ca^2+^ ions leading to unresponsiveness of cells toward NAADP (3, 26, 36, 37). In line with these results, in this study on ventricular cardiac myocytes the proton pump inhibitor bafilomycin A1 blocked both NAADP-mediated Ca^2+^ release in quiescent cardiac myocytes as well as SCT observed upon strong adrenergic stimulation. In addition, the NAADP antagonist BZ194 blocked SCT in a similar fashion as did bafilomycin A1. Thus, the present findings suggest an interconnection of NAADP-mediated Ca^2+^ release and sensitization of the global Ca^2+^ release machinery.

What is the present view of induction of SCT? The two major mechanisms involved in SCT generation in single cardiac myocytes are sensitization of RyR2 and an increase in luminal Ca^2+^ load of the SR (41, 42). Both mechanisms may be activated when Ca^2+^ release is induced *in addition* to the basic excitation-contraction machinery. Indeed, this was shown for endothelin-1-mediated generation of inositol 1,4,5-trisphosphate, where locally released Ca^2+^ from inositol 1,4,5-trisphosphate-sensitive stores led to SCTs (43). In line with this report, we demonstrate here that Ca^2+^ release from NAADP-sensitive stores leads to SCTs. Moreover, direct imaging of [Ca^2+^]_SR_ demonstrates spontaneous diastolic Ca^2+^ release events (determined as decrease of [Ca^2+^]_SR_) upon Iso stimulation; importantly, BZ194 prevented these spontaneous diastolic Ca^2+^ release events (Fig. 8). Collectively, these data suggest that strong β-adrenergic stimulation results in increased NAADP; exaggerated Ca^2+^ release from NAADP-sensitive stores strongly enhances the sensitivity of RyR2, and spontaneous global Ca^2+^ release by sensitized RyR2 is then visualized as SCT.

In previous studies in guinea pig ventricular myocytes, it was shown that β-adrenergic stimulation resulted in elevated endogenous NAADP (26, 44). Moreover, both caged NAADP or the membrane-permeant prodrug NAADP/AM increased the amplitude of Ca^2+^ transients and the amplitude of myocyte contraction (26). The authors hypothesized that the NAADP produced upon β-adrenergic stimulation resulted in additional Ca^2+^ release from acidic stores; this in turn would result in higher SR Ca^2+^ load, which was actually shown, and increased amplitudes of subsequent Ca^2+^ transients (26). In our model of mouse cardiac myocytes, we used strong β-adrenergic stimulation, well known not only to increase the amplitude and decay of Ca^2+^ transients but also to induce SCT (43, 45–48). Under these conditions, NAADP antagonism by BZ194, other than the intervention used in (26), did not affect the amplitude of Ca^2+^ transients and only slightly reduced the reciprocal time constant upon Iso addition. Instead, it completely and specifically suppressed SCTs, as discussed above. Based on these findings, we propose a profound and novel pathological role of NAADP in the generation of cellular arrhythmias induced upon strong β-adrenergic stimulation.

Corresponding to the inhibitory effect of NAADP antagonism on the development of SCT *in vitro,* we also observed a marked reduction of Iso-induced arrhythmias *in vivo*. Pretreatment of awake mice with BZ194 reduced the number of arrhythmic events after Iso injection by >90%. If we assume that the Iso-induced arrhythmic events *in vivo* were caused to a large extent by the cellular mechanisms observed in our *in vitro* studies in isolated cardiac myocytes (*i.e.* a significant increase in the occurrence of SCT), it appears likely that the antiarrhythmic effect of NAADP antagonism resulted from suppression of diastolic Ca^2+^ release from the SR. As detailed above, the magnitude of Ca^2+^ diastolic leak from the SR is critically dependent on the sensitivity of RyR2.

Unexpectedly, we observed signs of physical and behavioral impairment in mice that had received both Iso and BZ194, and about 50% of these animals died within a few days after treatment. The mechanisms leading to these severe side effects are currently unclear and may be explained by mechanism-related as well as off-target actions of the administered drugs. In preliminary experiments, we had tested the tolerability of an intraperitoneal injection of 180 mg/kg BZ194 in four mice, which all survived. This suggests that drug interactions between Iso and BZ194 might have played a major role in the pathogenesis of the observed high lethality. Clearly, the data indicate that BZ194 itself is unsuitable for treatment in human patients but do not argue against the proposed mechanism of action. In any case, further detailed pharmacological studies on the safety of BZ194 are mandatory.

In summary, a novel pathological role of NAADP in the generation of cellular arrhythmias induced upon strong β-adrenergic stimulation was discovered. We demonstrate full antagonism of SCT in cardiac myocytes *in vitro* and a high degree of antagonism of ventricular arrhythmogenic events *in vivo* by the NAADP antagonist BZ194 suggesting that the NAADP signaling pathway in cardiac myocytes may become suitable as a target for pharmaceutical intervention in cardiac arrhythmias.

## Footnotes

6 The abbreviations used are:

NAADP: nicotinic acid adenine dinucleotide phosphate
6-Bnz-cAMP: *N*^6^-benzoyladenosine-3′,5′-cyclic monophosphate
[Ca^2+^]*_i_*: free cytosolic Ca^2+^ concentration
[Ca^2+^]_SR_: sarcoplasmic reticular-luminal Ca^2+^ concentration
Iso: isoproterenol
8-pCPT: 8-(4-chlorophenylthio)-2′-*O*-methyladenosine-3′,5′-cyclic monophosphate
Epac: exchange protein activated by cAMP
PVB: premature ventricular beat
RyR: ryanodine receptor
SCT: spontaneous diastolic Ca^2+^ transient
SDC: spontaneous diastolic transient decreases in [Ca^2+^]_SR_
SERCA: sarcoplasmic/endoplasmic reticular Ca^2+^-ATPase
SR: sarcoplasmic reticulum
TRP-ML1: transient receptor potential channels, subtype mucolipin 1
VT: ventricular tachycardia
ANOVA: analysis of variance.
